# Going beyond conventional parameters to unveil sperm quality in fish: the use of fibre optic technology to assess mitochondrial respiratory performance

**DOI:** 10.1242/bio.053306

**Published:** 2020-07-31

**Authors:** Lisa Locatello, Giovanni Rigoni, Maria E. Soriano, Maria B. Rasotto, Federica Poli

**Affiliations:** Department of Biology, University of Padova, Via Ugo Bassi 58/b, 35121 Padova, Italy

**Keywords:** Fish, Sperm quality, Oxygen consumption rate, ATP, Sperm competition

## Abstract

Sperm fertilisation success depends on both intrinsic quality and the interactions with the surrounding reproductive fluids. In several fish species, these interactions have a variable effect on sperm performance. Although specific responses to reproductive fluids may depend on intrinsic differences in sperm quality, variations in the traditionally recorded sperm functional traits do not fully account for the observed patterns. New methods to enhance the evaluation of sperm quality may prove to be valuable at both applied and theoretical levels, by improving the breeding protocol of reared species and the understanding of mating success in sperm competition contexts. Here we develop a fibre optic-based technique, also adequate for small ejaculate samples, to test the role of mitochondrial respiratory efficiency in deciphering sperm performance variability. We purposely used as model the grass goby, *Zosterisessor ophiocephalus*, a fish with guard-sneaker mating tactics where the sperm in each male tactic have similar intrinsic qualities (velocity, viability, ATP content), but sneakers' sperm exploit territorial males' seminal fuid, overall displaying better fertilization ability. We found that sperm differed in their mitochondrial respiratory efficiency, which was higher in sneakers’ sperm compared to territorial ones. This result draws the attention to an indicator of sperm quality that might be helpful in disentangling the mechanisms driving sperm-reproductive fluid interactions.

## Abstract (precis)

**Summary:** A novel technique to measure sperm mitochondrial respiratory efficiency, an overlooked parameter of sperm quality that might contribute in unveiling mechanisms driving the intraspecific variability in sperm performance.

## INTRODUCTION

The number and quality of sperm are widely recognized to determine fertilisation success in both natural and artificial conditions (Birkhead and Møller, 1998; Birkhead et al., 2009; Morrell and Rodriguez-Martinez, 2009). Thus, one of the main goals in spermatology is to detect the set of sperm traits that allow a better prediction of fertilisation outcomes. In this respect, a major contribution to the field was engendered by studies on sperm competition, which is now recognized as a major selective force influencing both sperm morphology and physiology (Parker, 1970; Simmons and Fitzpatrick, 2012; Snook, 2005). Several of the assessed techniques and empirical results from this research have improved the theoretical understanding of the fertilisation outcomes in wild organisms, with important implications also in animal breeding and aquaculture (Morrell and Rodriguez-Martinez, 2009; Nordeide, 2007). Now, an ample body of evidence proves that an increase in the level of sperm competition is paralleled by a greater ejaculate investment in terms of sperm number and/or sperm quality traits, which are decisive to the fertilizing capacity, such as the size of different components (head, midpiece and flagellum) and the performance in terms of swimming speed, viability and longevity (Pizzari and Parker, 2009; Simmons and Fitzpatrick, 2012; Snook, 2005). Moreover, recent advances have shown that despite sperm performance having long been considered exclusively dependent on sperm intrinsic quality – meaning pure quality at the net of the non-sperm components of the ejaculate – sperm motility may be influenced by the composition of the reproductive fluids they interact with. Indeed, seminal fluid (SF), a conspicuous component of the ejaculate (Poiani, 2006), may affect either own and rival sperm velocity or viability (den Boer et al., 2010; Fry and Wilkinson, 2004; Lewis and Pitcher, 2017; Locatello et al., 2013; Poli et al., 2018) and males are able to modulate its composition in relation to sperm competition level, male status and female quality (Bartlett et al., 2017; Cornwallis and Birkhead, 2007; Locatello et al., 2013; Poli et al., 2018; Ramm et al., 2015; Simmons and Lovegrove, 2017; Wigby et al., 2009). Moreover, the female-derived fluid surrounding the eggs (i.e. the ovarian fluid, OF) may also influence sperm functional traits, such as activation, velocity, viability, longevity and swimming trajectory (for a review see Zadmajid et al., 2019). In several species, some of which are salmons of commercial value, such as the Arctic charr, *Salvelinus alpinus*, and the chinook salmon, *Oncorhynchus tshawytscha*, the OF effect on sperm performance varies depending on male and female identity (Elofsson et al., 2003; Gasparini and Pilastro, 2011; Rosengrave et al., 2008; Urbach et al., 2005). This male–female interaction effect has been suggested to partially rely on the superior intrinsic quality of the sperm released by some males, swimming faster or living longer in the presence of specific OF (Egeland et al., 2015; Poli et al., 2019; Rosengrave et al., 2008, 2009; Urbach et al., 2005). However, when sperm intrinsic quality is analysed, none of the usually recorded parameters (i.e. velocity, viability, longevity, ATP content, etc.) appear to fully explain the observed variability in the presence of reproductive fluids.

The mechanisms underlying the variation in fish sperm performance among sperm from different males activated in the same medium, whether rival male's SF or female's OF, are largely unknown. However, the most common response of sperm to reproductive fluid/s is a change in motility performance (Egeland et al., 2015; Lewis and Pitcher, 2017; Locatello et al., 2013; Poli et al., 2018; Rosengrave et al., 2009; Urbach et al., 2005). Sperm motility is mainly controlled by mitochondria, which have been suggested to be responsible for differences in sperm swimming speed among males (Froman and Kirby, 2005). In particular, mitochondrial respiratory efficiency strongly influences sperm motility and is a major determinant of fertility in humans (Amaral et al., 2013; Ferramosca et al., 2012), but, to our knowledge, it has never been recorded in sperm competition or cryptic female choice studies. In fish, the analysis of sperm respiration, a measure of mitochondrial function, has been restricted to larger species (e.g. goldfish, carp, turbot and cod) (Cosson, 2012), also due to methodological constraints imposed by conventional electrodes that require a sufficiently high amount of sperm. Moreover, this investigation has been limited to the basal oxygen consumption rate (Cosson, 2012), but a more informative measure of sperm mitochondria respiratory efficiency is represented in human and rodents by the spare sperm respiratory capacity, meaning the difference between the maximum respiratory capacity and the basal respiratory capacity (Dranka et al., 2011; Tourmente et al., 2015). The spare capacity indicates the amount of oxygen consumption that is available for cells to use during increased energy demand or other stress conditions (Tourmente et al., 2015).

Here we introduce a novel a technique to estimate sperm respiratory capacity in small amounts of fish ejaculate. This technique takes advantage of a microfibre optic oxygen meter to measure both the basal and the maximum oxygen consumption rate of sperm, in order to estimate the overall sperm respiratory capacity (i.e. difference between basal and maximum respiration). The maximum oxygen consumption rate of sperm was reachable by the addition of a potent protonophore, the carbonyl cyanide p-[trifluoromethoxy]-phenyl-hydrazone (FCCP), that promotes the ‘uncoupling’ between the rate of electron transport in the respiratory chain and the oxidative phosphorylation, with a consequent collapse of the proton gradient and disruption of the mitochondrial membrane potential (Park et al., 2002). As a result, electron flow through the electron transport chain is maximized in the attempt to maintain the membrane potential, and thus inducing the maximal oxygen consumption rate (Park et al., 2002).

To apply this technique, we took advantage of the well-known system of the grass goby, *Zosterisessor* *ophiocephalus*, in which a specific male response to reproductive fluids, possibly depending on male variability in sperm intrinsic quality traits, has been recorded (Locatello et al., 2013). The grass goby is a fish species with external fertilisation and guard-sneaker mating tactics (Scaggiante et al., 1999). During the breeding season, territorial males dig and defend their nest, court females and perform parental care to the eggs. Sneaker males parasitize the spawnings of territorial males (Mazzoldi et al., 2000; Scaggiante et al., 1999). Territorial males release viscous ejaculates (sperm trails) on the nest ceiling, where eggs are laid both before and during egg deposition (Mazzoldi et al., 2000; Scaggiante et al., 1999). These ejaculates slowly dilute in seawater, thus releasing active sperm (Scaggiante et al., 1999). Sneaker males enter inside a nest when spawning occurs and release their ejaculate in proximity to those of territorial males and to eggs (Mazzoldi et al., 2000). Territorial males’ ejaculates contain more SF and fewer sperm than those of sneakers (Mazzoldi et al., 2000; Scaggiante et al., 1999). However, when sperm are assayed in a saline solution or in a male's own SF, their velocity, viability and ATP content (parameters of sperm quality that are commonly analysed in fish and, thus, can be considered as the most conventional ones; reviewed in Kowalski and Cejko, 2019), do not vary with tactic (Locatello et al., 2007, 2013). However, the presence of rival SF changes the scenario, since SF differently affects the sperm performance of other males in terms of velocity and fertilisation success, in relation to the tactic adopted by males (Locatello et al., 2013). Indeed, the performance of territorial males’ sperm is negatively affected by the SF of sneaker males, while sneaker sperm cells exploit territorial male SF, overall displaying better fertilisation ability (i.e. leading to a higher proportion of fertilized eggs in *in vitro* fertilisation trials) (Locatello et al., 2013). This is why it has been hypothesized that a difference in sperm quality between the two male morphs, which does not emerge when analysing conventional parameters, lies in other unexplored sperm features. We explored if a hidden difference in the intrinsic quality of the sperm of the two grass goby male morphs, which might explain the ability of sneaker sperm to better exploit the territorial male SF, relies on sperm mitochondrial respiratory efficiency. While our primary focus was on mitochondrial respiratory efficiency, we also measured basal sperm velocity and ATP content (i.e. sperm in saline solution) in order to validate our previous results obtained in 2007 (Locatello et al., 2007) and 2013 (Locatello et al., 2013), showing that these parameters do not differ between territorial and sneaker males.

## RESULTS

### Repeatability of measurements

The method used to estimate sperm velocity in the grass goby was already well validated in previous studies yielding high repeatability (sperm curvilinear velocity, VCL: R>0.7) (see Locatello et al., 2007). In the present study, we measured the within-sample repeatability by repeating the analyses twice for each sample, for both ATP content and oxygen consumption rate. This was done on ten samples for ATP content measurement (ten out of the total 19 samples in which we performed the whole analyses) and on another ten sperm samples for the measurement of oxygen consumption rate. In this latter case, the repeatability was tested on a preliminary set of ten males that were not used in the following analyses. Measurements of ATP content yielded a high significant repeatability: R=0.926±0.068 s.e.; CI=0.75, 0.979 (*P*<0.001). Measurement of oxygen consumption rate yielded a more moderate, but also significant repeatability: R=0.595±0.223 s.e.; CI=0, 0.874 (*P*=0.026).

### Sperm velocity and ATP content

Results on basal sperm velocity, measured on a set of 17 territorial and 17 sneaker males, and on ATP content, measured on another set of seven territorial and 12 sneaker males, confirmed the previous findings obtained in this species (Locatello et al., 2007, 2013). Indeed, these parameters did not differ between territorial and sneaker males (Table 1; Fig. S1).

**Table 1.**
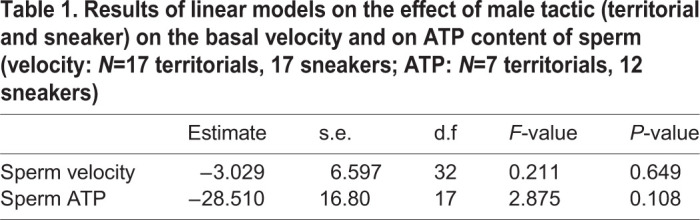
**Results of linear models on the effect of male tactic (territorial and sneaker) on the basal velocity and on ATP content of sperm (velocity: *N*=17 territorials, 17 sneakers; ATP: *N*=7 territorials, 12 sneakers)**

### Oxygen consumption rate

The oxygen consumption rate was measured on 21 sneaker and 20 territorial males, different from those in which we measured sperm velocity or ATP content.

**Fig. 1.**
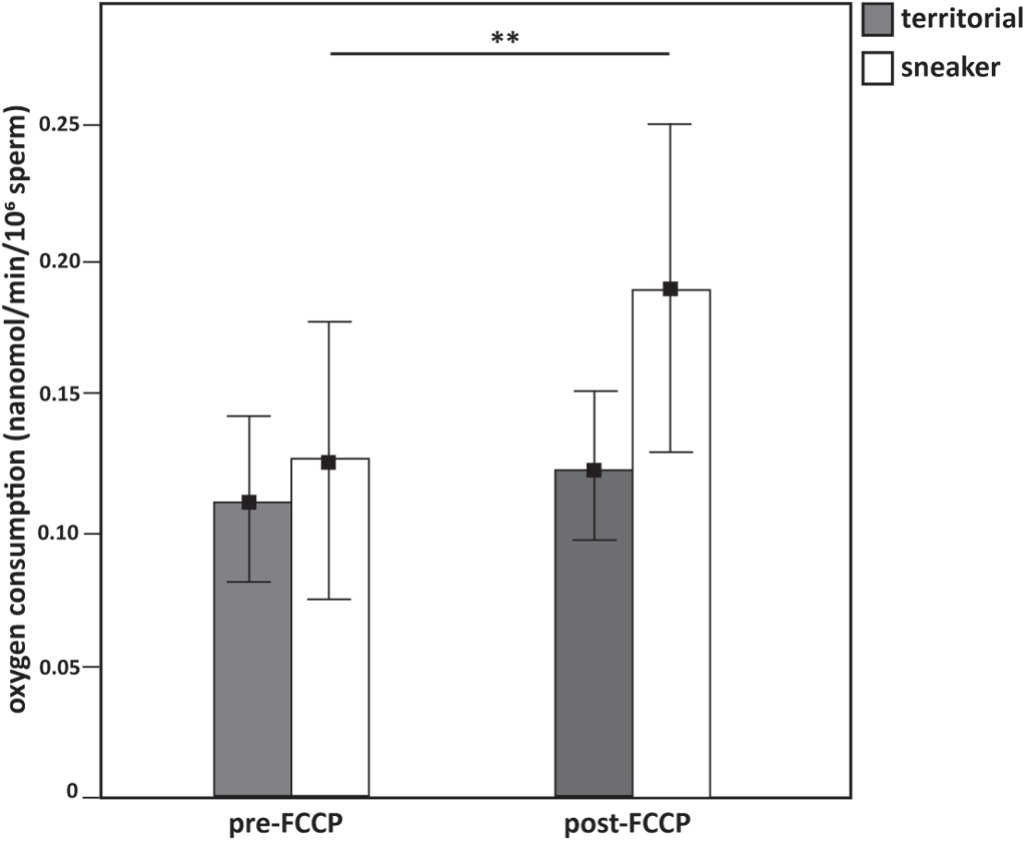
**Oxygen consumption rate measured in the first** **2** **min** **before and after the addition of FCCP in territorial and sneaker males' sperm (*N*=20 territorials, 21 sneakers).** Depicted is mean±confidence interval. ** adjusted *P*<0.01 (significant increase in oxygen consumption with the addition of FCCP in sneaker males' sperm. Post-hoc pairwise comparisons with Bonferroni correction).

The linear mixed effect model applied to test the effect of male tactic (territorial versus sneaker) and of the treatment (before the addition of FCCP versus after the addition of FCCP), showed a significant interaction between male tactic and treatment (Table 2), with a significantly higher increase of the oxygen consumption rate after the addition of FCCP in sneaker males compared to territorial ones (Fig. 1). Indeed, post-hoc comparisons of least squares means showed a significant increase in the oxygen consumption rate after the addition of FCCP only in sneaker males’ sperm and not in territorial ones (sneaker: estimate±s.e.=0.088±0.021, d.f.=39, t=4.287, adjusted *P*<0.001; territorial: estimate±s.e.=0.020±0.021, d.f.=39, t=0.954, *P*=1.000) (Fig. 1).

**Table 2.**
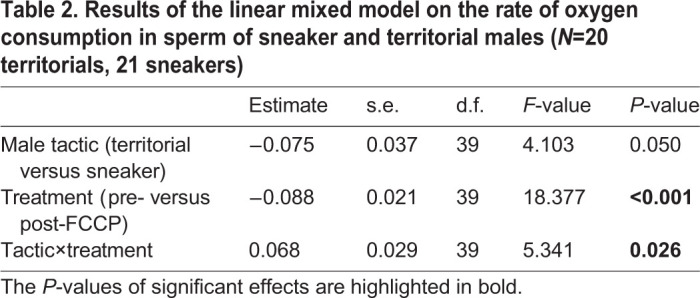
**Results of the linear mixed model on the rate of oxygen consumption in sperm of sneaker and territorial males (*N*=20 territorials, 21 sneakers)**

## DISCUSSION

The contributions of our study are manifold, on both the technical and the more general biological issues perspective. First, we introduced a method that, thanks to a fibre optic-based technology, allows the measurement of an overlooked sperm feature, the sperm oxygen consumption rate, in species in which the tiny amount of ejaculate represented a major limitation to the traditional measurements of respiration by means of electrodes. This limitation is quite common in sperm competition and cryptic female choice studies that are often performed in small model fish species (e.g. guppy, zebrafish) and insects. Moreover, the adjustment to the measuring chamber allowed going beyond the measurement of the basal sperm respiratory rate to analyse the actual mitochondrial respiratory efficiency, by stimulating the maximum oxygen consumption rate with the injection of a protonophore during the on-going measurement. This is an important step in improving the measurement of sperm functional traits, since the spare sperm respiratory capacity, meaning the difference between the maximum respiratory capacity and the basal respiratory capacity, actually represents the most exhaustive quantification of the mitochondrial respiratory efficiency (Dranka et al., 2011; Tourmente et al., 2015).

From a more general perspective, our study highlights the role of mitochondrial respiratory efficiency in evaluating sperm quality. Up until now, this sperm feature has been regularly used in human fertility analyses (see Ferramosca et al., 2012), but it was largely overlooked in the breeding protocols of reared species as well as in sperm competition and cryptic female choice studies. However, a male identity effect in response to reproductive fluids had been reported in several fish, including some salmons of commercial value (Elofsson et al., 2003; Firman et al., 2017; Gasparini and Pilastro, 2011; Poli et al., 2019; Rosengrave et al., 2008; Urbach et al., 2005). Excluding the cases in which the specific male–female interactions are mediated by genetic compatibility, such as in the guppy, *Poecilia reticulata* (Gasparini and Pilastro, 2011), the variability of sperm quality among males, not justified by differences in commonly recorded sperm traits, might rely on mitochondrial respiratory efficiency, allowing the sperm of some males to better exploit the OF over others (Egeland et al., 2015; Poli et al., 2019; Rosengrave et al., 2008, 2009; Urbach et al., 2005). Our study indicates the measure of mitochondrial respiratory efficiency as an addition parameter of interest that might also help in unravelling unexpected pattern of relationship among different sperm quality traits, such as the lack of functional association between sperm velocity and midpiece size often observed across different species (see Locatello et al., 2007). This pattern could be explained by higher sperm velocities linked to a higher mitochondrial respiratory capacity, rather than to a higher number or larger mitochondria (Anderson and Dixson, 2002; Cardullo and Baltz, 1991). Thus, as it already occurs in human fertility analysis, the inclusion of sperm mitochondrial respiratory capacity in the evaluation of ejaculate traits for artificial fertilisation might have major implications on the choice of brooders and on the set up of spawning protocols and breeding programs.

Finally, the newly developed technique applied to our model species, the grass goby, enabled us to highlight an otherwise hidden difference in the intrinsic sperm quality of males adopting different tactics. Indeed, sneakers’ sperm exhibit a higher respiratory capacity compared to territorial ones. In particular, when the mitochondrial chain is uncoupled by means of FCCP, the increase of oxygen consumption is higher in sneaker than in territorial males’ sperm. As demonstrated in human, sperm mitochondrial respiratory performance can be crucial in determining motility and fertilisation ability (Amaral et al., 2013; Ferramosca et al., 2012). Therefore, the observed differences in mitochondrial respiratory efficiency between the sperm released by the two grass goby male morphs help us to delve into the mechanism underlying the ability of grass goby sneaker sperm to take advantage of the territorial males' SF for enhancing their velocity and fertilisation rate (Locatello et al., 2013). This ability is not justified by commonly recorded sperm motility traits that do not differ between sneaker and territorial males’ sperm, with or without their own SF (Locatello et al., 2007, 2013; Scaggiante et al., 1999; present study). The present results suggest that the higher mitochondrial functionality of sneakers’ sperm might allow them to make the best of territorial males' SF, in increasing the supply of energy available by the flagellar beatings/time, therefore, resulting in a faster speed overall. Flagellar beat cross frequency, indeed, is related to the sperm propulsive energy and increased propulsion requires a higher rate of energy consumption (Butts et al., 2017; Cosson et al., 2008). Although the components of territorial males' SF responsible for the enhancement of sneaker sperm velocity remain unknown, the difference in sperm mitochondria functionality, between males adopting alternative mating tactics, sheds light onto the proximate mechanisms driving the tactic-dependent sperm-SF interaction occurring in this species. Overall, these findings represent an important step in the understanding of patterns of ejaculate investment in fish with male alternative reproductive tactics, suggesting that this new technique should be generally applied to species in which sperm competition occurs.

## MATERIALS AND METHODS

### Animal sampling and ejaculate collection

Males were collected in the Venetian Lagoon during their breeding season (April–June), anesthetized in a water solution of MS 222 (Tricaine sulphate, Sandoz) (0.5 g l^−1^), measured (SL: distance between the snout and the base of the tail), weighed (g) and categorized as a territorial or sneaker male. The tactic definition was based on males’ size and the characteristics of their sperm trails, which are white in sneaker males, owing to the high sperm content, and dense and opaque in territorial ones, owing to the high mucin content and low amount of sperm (Mazzoldi et al., 2000).

Male body condition (BC) was also calculated as weight×SL^−3^×100 (Bolger and Connolly, 1989). A total number of 94 males were used in this study (44 territorials, 50 sneakers), and different males were used in different assays (sample size for each assay is reported below). Ejaculate was collected with a Gilson pipette by gentle pressure on the abdomen of anesthetized males and centrifuged at 13.300× ***g*** for 3 min at 4°C to separate sperm from the supernatant SF. Sperm were then re-suspended in an inactivating medium (3.5 g l^−1^ NaCl, 0.11 g l^−1^ KCl, 0.39 g l^−1^ CaCl_2_, 1.23 g l^−1^ MgCl_2_, 1.68 g l^−1^ NaHCO_3_, 0.08 g l^−1^ glucose, pH 7.7) (Fauvel et al., 1999) and maintained at 3–5°C until analysis (within 1 h of collection). As the number of sperm varies among males and is significantly higher in sneakers than territorials, the volume of inactivating solution was individually adjusted (range: sneaker, 70–800 μl; territorial, 45–500 μl), in order to standardize for sperm concentration in inactivated samples (about 76,000 sperm/μl). Sperm concentration was checked with an improved Neubauer chamber haemocytometer.

### Sperm velocity and ATP content

Sperm velocity (curvilinear velocity VCL) was measured on 17 territorials (SL: 15.3–19.2 cm) and 17 sneakers (SL: 7.1–11.7 cm). Ten μl of sperm were taken from each inactivated sample and activated by adding 20 μl of filtered seawater at 20±1°C, containing 2 mg ml^−1^ of bovine serum albumin. Three μl of sample were then placed in separate wells on a 12-well multi-test slide (MP Biomedicals, Aurora, OH, USA) previously coated with 1% polyvinyl alcohol (Sigma-Aldrich), to avoid sperm sticking to the glass slide (Wilson-Leedy and Ingermann, 2007), and covered with a coverslip. Sperm velocity was measured using a CEROS Sperm Tracker (Hamilton Thorne Research, Beverly, MA, USA), at 60 fps. Mean speed measurements were based on 79±51 (mean±s.d.) sperm. We focused on curvilinear velocity (VCL, μm s^−1^), as this measure is a reliable predictor of the fertilisation success in many external fertilizers, including the grass goby (Casselman et al., 2006; Gallego et al., 2017; Locatello et al., 2013).

ATP content was measured in 2.5×10^6^ sperm from another set of seven territorials (SL: 16.7–20 cm) and 12 sneakers (SL: 7.4–11 cm), with a fluoroskan ascent FL Thermo Scientific, and following manufacturing protocols of the ATPlite Luminiscent Assay system (PerkinElmer). The standard curve was made by using the sperm resuspension buffer (Fauvel et al., 1999) and known increasing concentrations of ATP. Sperm concentration was checked with an improved Neubauer chamber haemocytometer and adjusted before each measurement. Results were reported as pmol/10^6^ sperm. In ten out of the total 19 males measurements were repeated twice to test the within sample repeatability, and mean values used for the analyses.

Territorial and sneaker males used for velocity and ATP analyses did not differ in their BC (linear model: velocity: estimate=−0.085, s.e.=0.051, t=−1.679, *P*=0.103; ATP: estimate=0.033, s.e.=0.045, t=0.715, *P*=0.485).

### Oxygen consumption rate

**Fig. 2.**
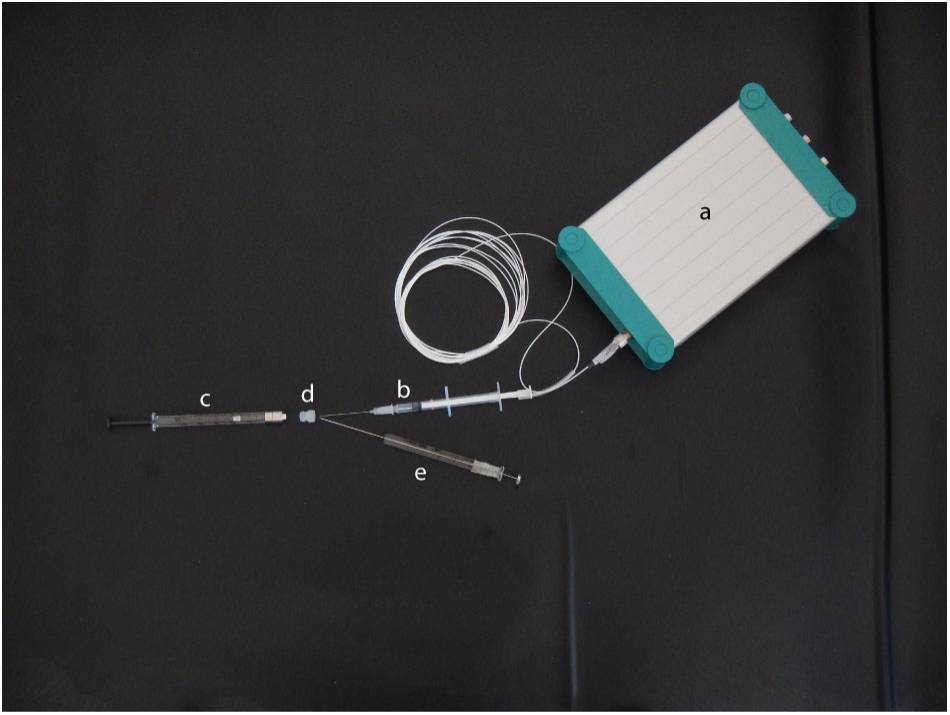
**Microfibre optic oxygen meter, Microx TX3 (A), equipped with a needle type optical.** Microsensor (B); gas-tight Hamilton syringe containing the sample (C) and sealed with a silicon pierceable cap (D); (E) Hamilton syringe used to inject FCCP during oxygen measurement by piercing the silicon cap (D).

Sperm oxygen consumption was measured with a microfibre optic oxygen meter, Microx TX3, equipped with a needle type optical microsensor (NTH-Pst1-L5-TF-NS40-0.4-YOP, PreSens, Germany) (Fig. 2) 2 min after sperm activation and 2 min after the addition of FCCP, in another set of 20 territorial (SL: 14.4–18.8 cm) and 21 sneaker (SL: 6–10.2 cm) males. Oxygen concentration was calculated from the value of oxygen partial pressure using temperature-dependent solubility coefficients for oxygen (αO_2_, μmol×l^−1^×Torr^−1^). Oxygen consumption rate was estimated by determining the decline in oxygen concentration over time, and was expressed as nanomol/min/10^6^ sperm. Prior to oxygen measurements, the sensor of the oxygen meter was calibrated in air-equilibrated seawater (100% oxygen saturation) and in sodium dithionite-saturated seawater (0% oxygen). Fifty μl of sperm in inactivating solution (Fauvel et al., 1999), containing a standard amount of 4×10^6^ sperm were activated with the addition of 100 μl of filtered seawater. Sperm concentration was checked with an improved Neubauer chamber haemocytometer and adjusted before each measurement. After 2 min of sperm activation, the solution was injected inside a gas-tight Hamilton syringe (1750TLL, 500 μl) (Fig. 2) using a microcapillary pipette tip, and the excess of air inside the syringe was expelled, leading to a final volume of 110 μl of sperm activated solution (=2.93×10^6^ sperm per sample). The syringe was sealed with a silicon pierceable cap and the needle microsensor (Fig. 2) inserted inside the syringe by piercing the cap. The optical fibre was extruded from the needle and the measurement of the basal oxygen consumption in the sample proceeded for 2 min. To reach the maximum oxygen consumption rate 4 µl of FCCP (150 µmol) were then added to the solution with a Hamilton syringe (702N, 25 µl) by further piercing the silicon syringe cap (Fig. 2). The measurement of oxygen by the fibre continued for another 2 min after the addition of FCCP. The difference between the FCCP-stimulated oxygen consumption rate and the basal oxygen consumption rate, measured before the addition of FCCP, yields an estimate of the sperm respiratory capacity. A syringe filled with only filtered marine seawater was used during each trial as a control, to account for background oxygen depletion. Measurements were always performed at 20°C room temperature. Territorial and sneaker males used to estimate oxygen consumption rate did not differ in their BC (linear model, estimate=−0.032, s.e.=0.051, t=−0.636, *P*=0.528).

In a preliminary set of ten males, measurements on basal oxygen consumption were repeated twice to test the within sample repeatability.

### Statistical analyses

All tests were performed using R Studio v 1.1.463. Repeatability of measurements was tested using the ‘rptR’ package, with Gaussian distribution and based on 1000 parametric bootstraps (Stoffel et al., 2017). Differences in basal sperm velocity and ATP content between territorial and sneaker males were analysed by linear models (‘lm’ function of the package ‘stats') (R Core team, 2019). The effect of male tactic (territorial versus sneaker), of treatment (pre-FCCP versus post-FCCP addition), and of their interaction on sperm oxygen consumption rate was investigated using a linear mixed model (‘lme’ function of the package ‘nlme’) (Pinheiro et al., 2017). Oxygen consumption, male tactic and treatment were considered as fixed factors, and male identity was included as a random factor, with the estimation of random intercepts for each subject, to account for repeated measures (before and after the addition of FCCP). All models yielded normally distributed residuals (Shapiro–Wilk normality test. Sperm velocity: W=0.967, *P*=0.382; sperm ATP content: W=0.917, *P*=0.098; sperm oxygen consumption: W=0.984, *P*=0.414 on square-root transformed data). Post-hoc pairwise comparisons for oxygen consumption rates were performed with the function ‘lsmeans’ (package ‘lsmeans’) (Lenth, 2016) and applying a Bonferroni correction for multiple comparisons.

### Ethics

Sampling and experimental procedures were approved by the animal ethics committee of the University of Padova (OPBA, permission no. 30/2015).

## Supplementary Material

Supplementary information
