# Supplementary material for: Going beyond conventional parameters to unveil sperm quality in fish: the use of fibre optic technology to assess mitochondrial respiratory performance
Source: Biol Open. 2020 Jul 31;9(7):bio053306. doi: 10.1242/bio.053306 (PMC7406323; doi:10.1242/bio.053306)
Supplement: Supplementary information [file biolopen-9-053306-s1.pdf]

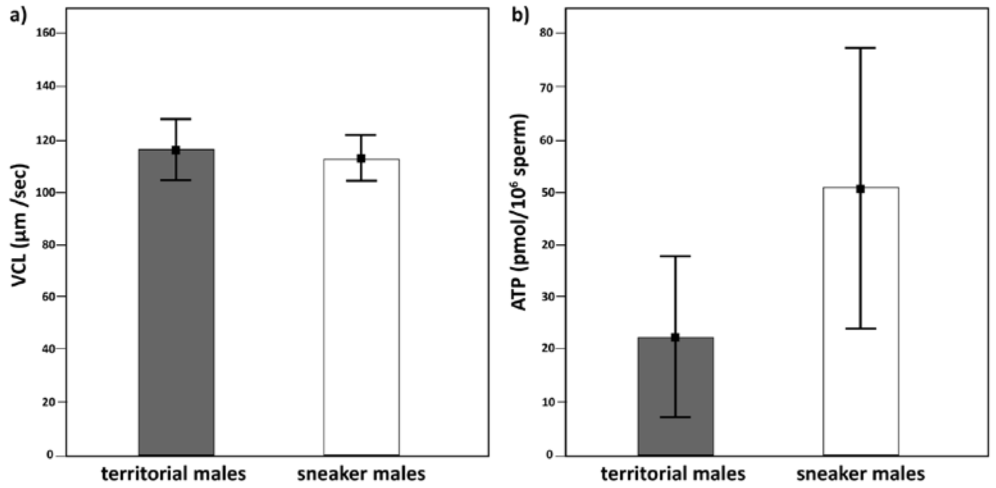

Fig. S1 a) curvilinear velocity (VCL) of territorial (n= 17) and sneaker (n=17) males' sperm. b) ATP content of territorial (n=7) and sneaker (n=12) males' sperm. Depicted is mean ± confidence interval.

Biology Open • Supplementary information
